# Construction of a preoperative emotional state and postoperative intra-abdominal pressure based prediction model for early enteral feeding intolerance in postoperative patients with gastric cancer

**DOI:** 10.3389/fnut.2024.1480390

**Published:** 2024-11-26

**Authors:** Yingying Xu, Qiongyuan Hu, Dandan Pei, Yin Zhang, Huanhuan Zhu, Yan Hui, Wenxian Guan, Meiling Xu, Li Chen

**Affiliations:** ^1^Division of Gastric and Hernia Surgery, Department of General Surgery, Nanjing Drum Tower Hospital, The Affiliated Hospital of Nanjing University Medical School, Nanjing, China; ^2^Department of Urology, Nanjing Drum Tower Hospital, Affiliated Hospital of Nanjing University Medical School, Nanjing, China

**Keywords:** gastric cancer, enteral nutrition, feeding intolerance, predictive model, Lasso regression

## Abstract

**Background:**

The incidence of enteral feeding intolerance (ENFI) in the early postoperative period is high in patients after gastric cancer resection due to the characteristics of surgical traumatic stress and changes in the physiological structure of the digestive tract, and the current evaluation of ENFI after gastric cancer resection mostly depends on the symptoms and complaints of patients after gastric cancer resection, which is lagging and subjective. Early accurate and objective prediction of the risk of early ENFI after gastric cancer resection is critical to guide clinical enteral nutrition practice.

**Materials and methods:**

This study included 470 patients who underwent radical gastric cancer surgery at the Division of Gastric Surgery of a tertiary hospital in Nanjing, Jiangsu Province, between November 2021 and October 2022. The patients were divided into a training set (*n* = 329) and a validation set (*n* = 141) in a 7:3 ratio. The predictors were first screened through Lasso regression. Subsequently, multifactorial logistic regression analysis was used to establish a model for predicting patients' early ENFI column charts after gastric cancer resection. Internal and external validation of the model were performed on the training set and validation set data, respectively, including plotting the area under the curve (AUC) of the receiver operating characteristic (ROC) curve and calibration curves to assess the differentiation and calibration of the prediction model. The Hosmer-Lemeshow test was also used to assess the fit of the model.

**Results:**

The incidence of early ENFI in postoperative patients with gastric cancer was 44.68% in the training set and 43.97% in the validation set. The final predictors entered into the model were enteral nutrition solution type (OR1 = 1.31/OR2 = 7.23), preoperative enteral nutrition pre-adaptation technique (OR = 0.29), surgical approach (OR = 2.21), preoperative Profile of Mood State-Short Form score (OR = 5.07), and intra-abdominal pressure (OR = 6.79). In the internal validation, the AUC was 0.836, the 95% CI ranged from 0.792 to 0.879, the Hosmer-Lemeshow test showed χ ^2^ = 4.368 and *P* = 0.737, the sensitivity was 0.775, and the specificity was 0.741. In the external validation, the AUC was 0.853, the 95% CI ranged from 0.788 to 0.919, the Hosmer-Lemeshow test showed χ^2^ = 13.740 and *P* = 0.089, the sensitivity was 0.785, and the specificity was 0.823.

**Conclusions:**

The Nomogram model of early ENFI in postoperative patients with gastric cancer, constructed on the basis of Lasso-logistic regression, had good predictive efficacy and may serve as a reference for healthcare professionals to identify high-risk patients with early ENFI after gastrectomy.

## 1 Introduction

Early initiation of enteral nutrition (EEN) after gastrectomy and achievement of the target feeding volume (25–30 kcal/d) are key transition periods for successful implementation of long-term enteral nutrition support (1). However, factors such as traumatic stress due to gastrectomy and changes in the physiological structure of the digestive tract can lead to transient gastrointestinal tract dysfunction in the early postoperative period, thus resulting in greater incidence of feeding intolerance (FI) symptoms, such as abdominal distension, nausea, and vomiting, during early postoperative enteral nutrition than observed in other diseases treated with surgery (3%−45.4%) (2). Consequently, feeding interruption frequently occurs, thereby hindering successful implementation of long-term postoperative enteral nutrition. At present, the assessment of ENFI after gastric cancer resection is driven primarily by clinicians' opinions and relies on patients' symptomatic manifestations and complaints after receiving EN. This assessment is subjective and may introduce lags, thus substantially affecting the efficiency of EEN implementation. Therefore, early accurate and objective prediction of the risk of early ENFI after gastric cancer resection is critical to guide clinical enteral nutrition practice and ensure smooth implementation of EEN. Although a risk prediction model of ENFI in critically ill patients and other surgical patients has been reported, its main predictors are not applicable to patients after gastric cancer resection. Patients with gastric cancer experience different degrees of emotional distress related to cancer diagnosis and treatment at different disease stages (3). Several studies have shown that emotions affect gastrointestinal function through the body's autonomic nervous system (ANS) and the neuroendocrine system (4, 5). The primary mechanism (6, 7) is that prolonged emotional stress leads to persistent activation of the autonomic nervous system (ANS), especially the sympathetic nervous system, resulting in increased release of stress hormones such as adrenaline and noradrenaline. These hormones not only raise heart rate and blood pressure but also affect gastrointestinal function by promoting smooth muscle contraction in the GI tract and reducing blood flow to the digestive system. Meanwhile, ENFI is an external response to gastrointestinal dysfunction (8). Thus, there is an inherent connection between emotional states and ENFI. Moreover, although several studies have demonstrated that abdominal hypertension is an independent risk factor for ENFI (9), detailed research on its predictive value for enteral nutrition intolerance in perioperative gastric cancer patients remains limited. Therefore, this study was aimed at constructing a risk prediction model for early ENFI in patients after gastric cancer resection, including preoperative mental state and postoperative intra-abdominal pressure as the main predictors, to provide an objective basis for early clinical decision-making.

## 2 Materials and methods

### 2.1 Study design and patient enrollment

Patients who underwent radical gastrectomy for gastric cancer at the gastric surgery department of a tertiary hospital in Nanjing, Jiangsu Province, China, between November 2021 and October 2022 were included. The **inclusion criteria were as follows:** (1) patients ≥18 years of age, pathologic diagnosis of gastric cancer, with diagnostic criteria following the “Gastric Cancer Diagnosis and Treatment Guidelines, 2018 Edition” (10); (2) patients who underwent gastrectomy for gastric cancer; (3) patients who received postoperative post-pyloric intestinal feeding; and (4) patients with no previous mental illnesses, such as anxiety, depression, or other psychological diseases. **The exclusion criteria were as follows:** (1) patients with a history of previous tumors; (2) patients with concomitant functional intestinal lesions leading to gastrointestinal dysfunction (e.g., long-term diarrhea); (3) patients with postoperative pathological diagnosis of non-gastric cancers, including low-grade intraepithelial neoplasia, high-grade intraepithelial neoplasia, or gastric mesenchymal stromal tumors; (4) patients who underwent a second surgery because of early complications after the initial surgery; and (5) patients with concomitant serious underlying diseases, such as chronic enteritis or Crohn's disease. Rejection criteria **included** (1) patients with unplanned extubation of enteral feeding; (2) patients with serious postoperative complications during the course of the disease, such as active bleeding in the digestive tract, anastomotic fistula, intra-abdominal infection, and other contraindications to enteral nutrition. The study received ethical approval by the hospital ethics committee (ethics No. 2022-203-01). All patients or their family members provided signed informed consent. **Sample size:** The sample size in the modeling cohort was calculated with the method logistics independent variable event number [events per variable (EPV)]. When the Wald method was used, the EPV was greater than 5 to ensure stable results. The number of risk factors initially investigated in this study was 23 (Table 1). According to the literature (11) the incidence of ENFI after gastrectomy for gastric cancer can be 49.3%. In addition, sample loss of 10%−20% was considered. Therefore, the sample size required for the present study was determined to be (23 × 5 ÷ 0.9) ÷ 0.493 = 259 cases. The sample size in the modeling group in this study was 329 cases.

**Table 1:** Factors affecting ENFI in the early postoperative period after gastric cancer resection.

**Category**	**Independent variable**	**Assignment method**	**Data collection method**
General information	Gender	Male = 1; Female = 2	Collected on admission
	Age (yr)	< 60 = 1; ≥60 = 2	
	BMI	< 18.5 = 1; 18.5 to < 24 = 2; 24 to < 28 = 3; ≥28 = 4	
	Diabetes	No = 0; Yes = 1	
	Hypertension	No = 0; Yes = 1	
Information on specialist diseases	History of abdominal surgery	No = 0; Yes = 1	Collected on admission
	Preoperative NRS-2002 score	< 3 = 1; 3–5 = 2; ≥5 = 3	
	Preoperative PG-SGA score	0–1 = 1; 2–3 = 2; 4–8 = 3; ≥9 = 4	
	History of preoperative constipation	No = 0; Yes = 1	
	Preoperative neoadjuvant therapy	No = 0; Yes = 1	
	Preoperative POMS-SF score	< 27.5 = 1; ≥27.5 = 2	
	Pre-adaptive techniques for preoperative enteral nutrition	No = 0; Yes = 1	As recommended by the nutritional therapist
Surgery information	Surgical procedures for stomach cancer	Proximal = 1; Distal BiI = 2; Distal BiII = 3; Midgastric = 4; Total = 5	Surgical transcripts
	Surgical procedure	Open *=* 1; laparoscopic = 2	
	Intraoperative bleeding	< 400 ml = 1; ≥400 = 2	
	Surgical time	< 210 = 1; ≥210 = 2	
	Clinical staging	0 = 1; I = 2; II = 3; III = 4; IV = 5	Pathological report
	Postoperative ICU admission	No = 0; Yes = 1	Specialist record sheets
Information on the first postoperative day	ALB	< 40 = 1; ≥40 = 2	Laboratory indicators
	Anemia after surgery	No = 0; mild = 1; moderate = 2	
	K^+^	< 3.5 = 1; 3.5–4.2 = 2; ≥4.2 = 3	
	Intra-abdominal pressure before enteral feeding	< 12 = 1; ≥12 = 2	Bedside measurement of intra-abdominal pressure
	Type of enteral nutrition solutions used	S*P =* 1; T*P =* 2; TF*P =* 3	As recommended by the nutritional therapist

POMS-SF, Profile of Mood State-Short Form; SP, short peptide enteral nutrition; TP, total protein enteral nutrition; TFP, total protein with fiber enteral nutrition.

### 2.2 Outcome measures and data collection

#### 2.2.1 Criteria for determining ENFI

The European Society of Intensive Care Medicine (ESICM) clarified the definition of ENFI in 2012, referring to inability to achieve 83.68 kJ through the tube-feeding route within 72 h of initiation of enteral nutritional support or when EN is interrupted because of the development of gastrointestinal intolerance or other clinical reasons. The definition is symptoms of gastrointestinal intolerance during the process of EN, manifesting primarily as nausea, vomiting, diarrhea, abdominal distension, constipation, absent or weak intestinal sounds, and gastric retention greater than 1,000 mL/24 h. Patients who undergo gastrectomy for gastric cancer are at high risk of high intra-abdominal hypertension. At present, most patients in clinical practice receive trophic enteral feeding (12), and most have difficulties in achieving the target feeding amount within 72 h. Therefore, in this study, we continuously and dynamically monitored intolerance to enteral nutrition feeding 1–3 d postoperatively and considered interruption of feeding due to gastrointestinal intolerance symptoms during 1–3 d of postoperative enteral feeding as the criterion for determining early ENFI after gastrectomy in patients with gastric cancer.

#### 2.2.2 Methods of intra-abdominal pressure monitoring

The World Society of Abdominal Compartment Societies (WSACS) uses bladder pressure as a standard measurement of intra-abdominal pressure (13), which is performed as follows: the patient is placed in the supine position, a Foley catheter is used, and 25 mL sterilized saline is injected after bladder emptying. A hydrometer is connected with a three-way connector, with the mid-axillary line as the zero plane. The height of the water column at the end of the patient's expiration is the intra-abdominal pressure, which is re-measured 3 min after the first measurement, and the average of the two measurements is obtained. In this study, bladder pressure was measured by responsible nurse before enteral feeding during the 24 h postoperative period and was converted to the intra-abdominal pressure value by measurement of the height of the water column. A sustained increase in intra-abdominal pressure >12 mmHg is considered intra-abdominal hypertension (13); therefore, the threshold of intra-abdominal pressure in this study was 12 mmHg.

#### 2.2.3 Assessment of preoperative mental state

The Chinese version of the Profile of Mood State-Short Form (POMS-SF) was used to assess patients' mental state. The scale consists of 30 adjectives for self-assessment of mental state in the past week, and is divided into six dimensions: tension-anxiety, depression-frustration, anger-hostility, fatigue-sluggishness, confusion-confusion, and energy-vitality. Scoring uses a Likert 5-point scale, where 0 represents none, and 4 represents very much; energy-vitality is the positive mood dimension, whereas the other dimensions are negative (14). Cronbach's alpha coefficient of this scale is 0.67–0.93, and the critical value was 27.5, according to Zengzeng and Weili (15). The POMS-SF was administered in the preoperative study of patients undergoing radical gastric cancer surgery.

#### 2.2.4 Identification of other clinical predictors

Nine specialists are engaged in gastrointestinal surgery, gastroenterology, nutrition, and critical care medicine were invited to conduct an expert meeting for validation “The Questionnaire on Factors affecting ENFI in the early postoperative period after gastric cancer resection and their Assigning Values.” The response rate of the participating experts was 100%, and the degree of authority Cr was (Ca+Cs)/2 = (0.86 + 0.82)/2 = 0.84, thus indicating high authority and credibility. This questionnaire consisted of four parts, as shown in Table 1: (1) General information: gender, age, body mass index (BMI), diabetes mellitus, and hypertension; (2) Information on specialist diseases: history of abdominal surgery, preoperative NRS-2002 score, preoperative PG-SGA score, preoperative history of constipation, preoperative neoadjuvant therapy, preoperative POMS-SF score, and preoperative enteral nutritional pre-adaptation techniques; (3) surgical situation: gastric cancer surgery modality, surgical approach, intraoperative bleeding, duration of surgery, clinical stage, and ICU admission after surgery; (4) postoperative status in the first day: albumin (ALB) level, whether anemia was present in the postoperative period, potassium (K+), intra-abdominal pressure before enteral feeding, and type of enteral nutrition solution used.

Among these factors, preoperative enteral nutrition pre-adaptation technology was used for preoperative oral enteral nutrition in the preoperative period at our research center. The specific implementation process was as follows: patients underwent preoperative nutritional risk screening by nutritional pharmacists using the NRS-2002, malnutrition assessment with the PG-SGA, and for patients with no risk of malnutrition (NRS-2002 < 3 points, PG-SGA < 2 points) and Patients with no contraindications to enteral nutrition (e.g., intestinal obstruction, severe shock, localized intestinal ischemia, etc.) were given oral enteral nutrition solution 3D before surgery. The specific type of oral nutrition solution was decided upon by the nutritional pharmacist according to patient condition.

### 2.3 Statistical analysis

SPSS 25.0 was used for data analysis. Categorical variables are expressed as frequencies and percentages. Comparisons between groups were performed with the χ^2^ test or Fisher's exact test, and the rank sum test was used for hierarchical information. Lasso regression analysis was performed with the glmnet package in R4.1.0 to screen variables. The 10-fold cross-validation method was used for validation, and Lambda (λ) = Lambda 1se was set as the defining criterion for screening variables. Logistic regression analysis was performed with the rms software package and transformed into visualized column line graphs. The predictive efficacy of the model was assessed with receiver operating characteristic (ROC) analysis, calibration curves, and the Hosmer-Lemeshow test. *P* < 0.05 was statistically significant.

## 3 Results

### 3.1 Patient characteristics

Our research center implemented medical care support for patients in strict accordance with gastric cancer diagnosis and treatment protocols, including preoperative nutritional risk assessment/screening/diagnosis/intervention, correction of preoperative abnormal indicators (hypoproteinemia, hypertension, hypokalemia, anemia, etc.), placement of a nasoenteric tube for post-pyloric enteral feeding during the operation, postoperative multimodal analgesia and development of a standardized postoperative rehabilitation program based on factors including pain level assessment and clinical recovery indicators, to ensure comparable baseline levels across participants. A total of 470 patients were included in this study and were randomly divided into a training set and validation set in a 7:3 ratio. In the training set, 329 patients had a total of 147 cases of ENFI, with an incidence rate of 42.86%, whereas in the validation set, 141 patients had a total of 62 cases of ENFI, with an incidence rate of 43.97%. A total of 45 patients received preoperative neoadjuvant therapy, including 29 in the training cohort and 16 in the validation cohort. The median time from the end of neoadjuvant therapy to surgery was 21 (19, 25) days in the training cohort and 21.5 (20, 25) days in the validation cohort. Patients receive neoadjuvant therapy including SOX, SOX plus anti-PD1 antibody, S-1 plus nab-paclitaxel, or XELOX. In the training cohort, 9 patients received the SOX regimen, 16 received the SOX plus anti-PD1 regimen, and 3 received the S-1 plus Nab-PTX regimen, with a treatment duration of 6 (6, 6). In the validation cohort, 9 patients received the SOX regimen, 6 received the SOX plus anti-PD1 regimen, and 1 received the S-1 plus Nab-PTX regimen, with a treatment duration of 6 (6, 6). The time of hospitalization for patients who developed ENFI postoperatively in the training cohort was 16 (14, 17) days, and for those who did not develop ENFI, it was 15 (13, 17) days. In the validation cohort, the hospital stay for patients who developed ENFI was 15 (14, 18) days, and for those who did not, it was 14 (13, 16) days. A comparison of general information between the modeling set and the validation set is shown in Table 2.

**Table 2:** Comparison of general information between the training and validation sets.

**Variable**	**Variable level**	**Training set (*N* = 329)**	**Validation set (*N* = 141)**	**Statistical value**	***P***
ENFI	No	182 (55.3%)	79 (56.0%)	0.002	0.968
	Yes	147 (44.7%)	62 (44.0%)		
BMI (kg/m^2^)	< 18.5	10 (3.0%)	2 (1.4%)	1.161	0.762
	18.5 to < 24	195 (59.3%)	86 (61.0%)		
	24 to < 28	103 (31.3%)	43 (30.5%)		
	≥28	21 (6.4%)	10 (7.1%)		
Age (yr)	< 60	113 (34.3%)	47 (33.3%)	0.011	0.915
	≥60	216 (65.7%)	94 (66.7%)		
Intraoperative blood loss (mL)	< 400	314 (95.4%)	132 (93.6%)	0.353	0.552
	≥400	15 (4.6%)	9 (6.4%)		
Surgical time (min)	< 210	161 (48.9%)	73 (51.8%)	0.214	0.643
	≥210	168 (51.1%)	68 (48.2%)		
Albumin (g/L)	< 40	161 (48.9%)	63 (44.7%)	0.556	0.456
	≥40	168 (51.1%)	78 (55.3%)		
K^+^ (mmol/L)	< 3.5	17 (5.2%)	11 (7.8%)	3.746	0.154
	3.5–4.2	158 (48.0%)	55 (39.0%)		
	≥4.2	154 (46.8%)	75 (53.2%)		
Preoperative NRS-2002 (points)	< 3	123 (37.4%)	55 (39.0%)	0.255	0.880
	3–5	122 (37.1%)	53 (37.6%)		
	≥5	84 (25.5%)	33 (23.4%)		
PG-SGA (points)	0–1	22 (6.7%)	6 (4.3%)	1.045	0.790
	2–3	80 (24.3%)	35 (24.8%)		
	4–8	131 (39.8%)	58 (41.1%)		
	≥9	96 (29.2%)	42 (29.8%)		
POMS-SF (points)	< 27.5	221 (67.2%)	91 (64.5%)	0.200	0.655
	≥27.5	108 (32.8%)	50 (35.5%)		
Intra-abdominal pressure before enteral feeding (mmHg)	< 12	300 (91.2%)	122 (86.5%)	1.857	0.173
	≥12	29 (8.8%)	19 (13.5%)		
Type of enteral nutrition solution	SP	70 (21.3%)	28 (19.9%)	0.161	0.923
	TP	130 (39.5%)	58 (41.1%)		
	TFP	129 (39.2%)	55 (39.0%)		
Clinical staging of the disease	0	1 (0.3%)	0 (0.0%)	1.609	0.807
	I	103 (31.3%)	49 (34.8%)		
	II	71 (21.6%)	31 (22.0%)		
	III	136 (41.3%)	56 (39.7%)		
	IV	18 (5.5%)	5 (3.5%)		
Gastrectomy	Proximal	23 (7.0%)	6 (4.3%)	2.469	0.650
	Distal BiI	4 (1.2%)	1 (0.7%)		
	Distal BiII	128 (38.9%)	58 (41.1%)		
	Midgastric	6 (1.8%)	1 (0.7%)		
	Total	168 (51.1%)	75 (53.2%)		
Gender	Male	233 (70.8%)	101 (71.6%)	0.004	0.947
	Female	96 (29.2%)	40 (28.4%)		
High blood pressure	No	210 (63.8%)	85 (60.3%)	0.390	0.532
	Yes	119 (36.2%)	56 (39.7%)		
Diabetes	No	277 (84.2%)	117 (83.0%)	0.037	0.848
	Yes	52 (15.8%)	24 (17.0%)		
History of constipation	No	313 (95.1%)	136 (96.5%)	0.152	0.697
	Yes	16 (4.9%)	5 (3.5%)		
Preoperative neoadjuvant therapy	No	300 (91.2%)	125 (88.7%)	0.468	0.494
	Yes	29 (8.8%)	16 (11.3%)		
Postoperative transfer to ICU	No	247 (75.1%)	104 (73.8%)	0.034	0.853
	Yes	82 (24.9%)	37 (26.2%)		
Surgical procedure	Open	150 (45.6%)	69 (48.9%)	0.319	0.572
	Laparoscopy	179 (54.4%)	72 (51.1%)		
History of abdominal surgery	No	282 (85.7%)	125 (88.7%)	0.503	0.478
	Yes	47 (14.3%)	16 (11.3%)		
Anemia	No	207 (62.9%)	89 (63.1%)	4.969	0.083
	Mild	117 (35.6%)	45 (31.9%)		
	Moderate	5 (1.5%)	7 (5.0%)		
Pre-adaptive techniques for preoperative enteral nutrition	NO	240 (72.9%)	109 (77.3%)	0.765	0.382
	YES	89 (27.1%)	32 (22.7%)		
Hospital stay (days)	ENFI	16 (14, 17)	15 (14, 18)	−0.347	0.728
	NO ENFI	15 (13, 17)	14 (13, 16)	−1.869	0.062

### 3.2 Lasso regression analysis of early ENFI in patients after gastric cancer resection

**Figure 1:**
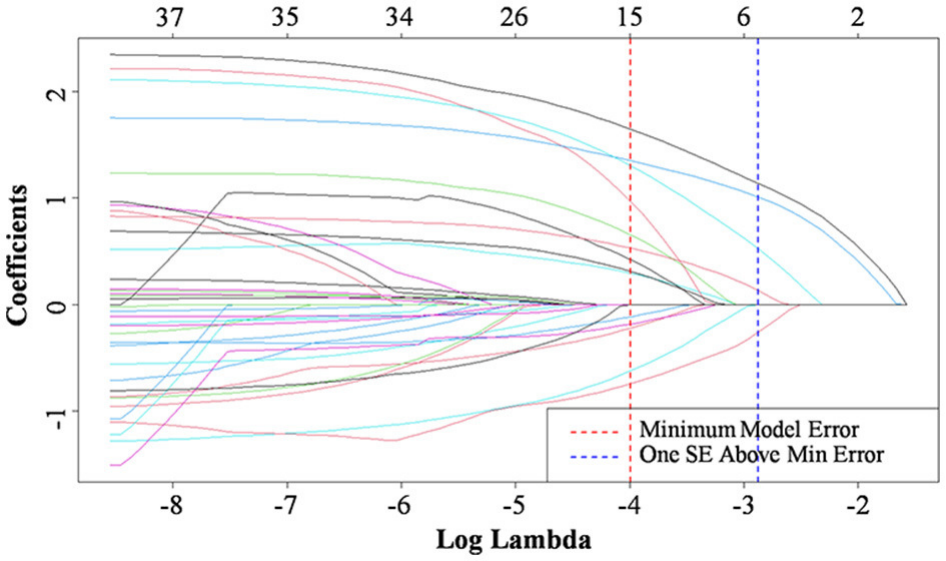
Characteristic of variable coefficient changes. Selection of predictive variables using Lasso regression. The horizontal coordinates represent the value of the parameter log(λ) ordinate represents the coefficient of the independent variable. Finally, the coefficients of all independent variables are compressed to 0, and the later the independent variable becomes 0, the greater the contribution to the model. The red dashed line indicates the vale of the parameter log(λ) when the model error is minimal, the blue dashed line represents the value of the parameter log(λ) when the model error is amplified by one standard error. The five lines to the right of the blue dashed line are the final selected five predictors: Pre-adaptive techniques for preoperative enteral nutrition, Surgical procedure, POMS-SF score, intra-abdominal pressure before enteral feeding, and type of enteral nutrition solution.

**Figure 2:**
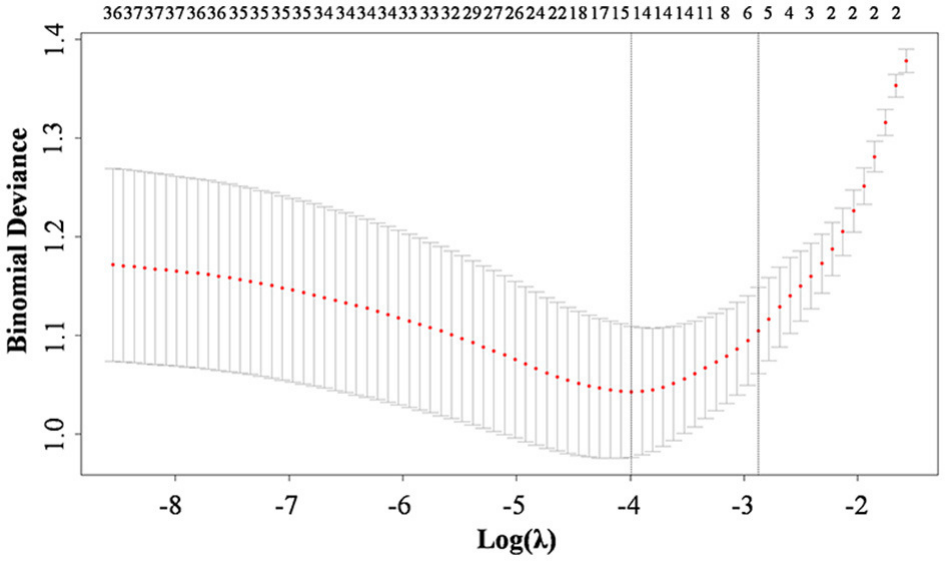
Ten-fold cross-validation for adjusting parameters. The horizontal coordinates represent the value of the parameter log (λ), ordinate represents the binominal deviance of the model.

Lasso regression analysis was performed with the above indicators as independent variables and ENFI as the dependent variable. The results of the Lasso regression are shown in Figure 1. Variables with non-zero coefficients were extracted through derivation of the optimal lambda. The dynamic process of Lasso regression screening of the variables is shown in Figure 1. When lambda (λ) increased, the coefficients of the initially included variables gradually compressed until they were 0 and eliminated. The process of cross-validation selection is shown in Figure 2. When λ had the value of lambda.min with the smallest model estimation error, the number of variables filtered out is 13, whereas when λ had the value of lambda 1se with the largest error within 1 standard deviation, The number of variables filtered out is 5. To avoid overfitting, and to identify the optimal penalty-order coefficients, and make the model streamlined and easy to use in clinical practice, we included the following five predictors: Pre-adaptive techniques for preoperative enteral nutrition, Surgical procedure, POMS-SF score, intra-abdominal pressure before enteral feeding, and type of enteral nutrition solution.

### 3.3 Multivariate analysis of early ENFI in patients after gastric cancer resection

**Figure 3:**
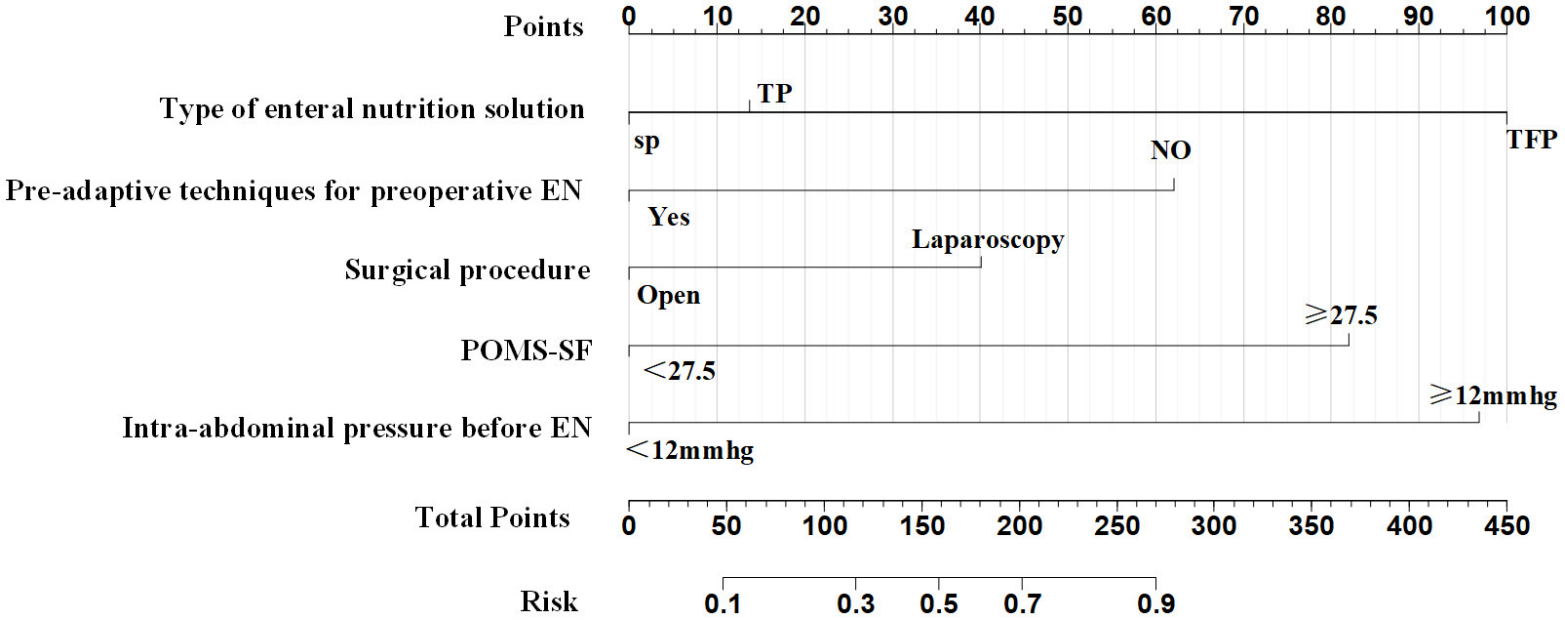
Nomogram for predicting whether patients undergoing Gastric Cancer Resection with ENFI.

The five predictors screened with Lasso regression were subjected to multifactorial logistic regression analysis to derive the regression coefficients of each predictor variable, as shown in Table 3, in which Pre-adaptive techniques for preoperative enteral nutrition, Surgical procedure, POMS-SF score, intra-abdominal pressure value before enteral feeding, and type of enteral nutrient solution were the independent predictor of ENFI in patients who had undergone gastric cancer resection (*P* < 0.05). A nomogram graph was additionally constructed (Figure 3).

**Table 3:** Logistic regression analysis of early enteral nutrition intolerance after gastric cancer resection.

**Regression coefficient**	**β**	**SE**	**Ward**	***P***	**OR**	**95%CI**	
						**Lower limit**	**Limit**
Continuous	−1.91	0.37	−5.20	< 0.001	-	-	-
**Pre-adaptive techniques for preoperative enteral nutrition**							
No (ref)	-	-	-	-	-	-	-
Yes	−1.22	0.34	−3.63	0.003	0.29	0.15	0.57
**Surgical procedure**							
Open (ref)	-	-	-	-	-	-	-
Laparoscopy	0.79	0.29	2.74	0.006	2.21	1.25	3.90
**POMS-SF (points)**							
< 27.5 (ref)	-	-	-	-	-	-	-
≥27.5	1.62	0.31	5.30	< 0.001	5.07	2.78	9.24
**Intra-abdominal pressure before enteral feeding (mmHg)**							
< 12 (ref)	-	-	-	-	-	-	-
≥12	1.92	0.64	3.01	0.003	6.79	1.95	23.63
**Type of enteral nutrition solution**							
SP (ref)	-	-	-	-	-	-	-
TP	0.27	0.39	0.70	0.48	1.31	0.61	2.80
TFP	1.98	0.39	5.14	< 0.001	7.23	3.40	15.37

POMS-SF, Profile of Mood State-Short Form; SP, short peptide enteral nutrition; TP, total protein enteral nutrition; TFP, total protein fiber enteral nutrition.

### 3.4 Validation of the risk prediction model for early ENFI after gastric cancer resection

#### 3.4.1 Internal validation

**Figure 4:**
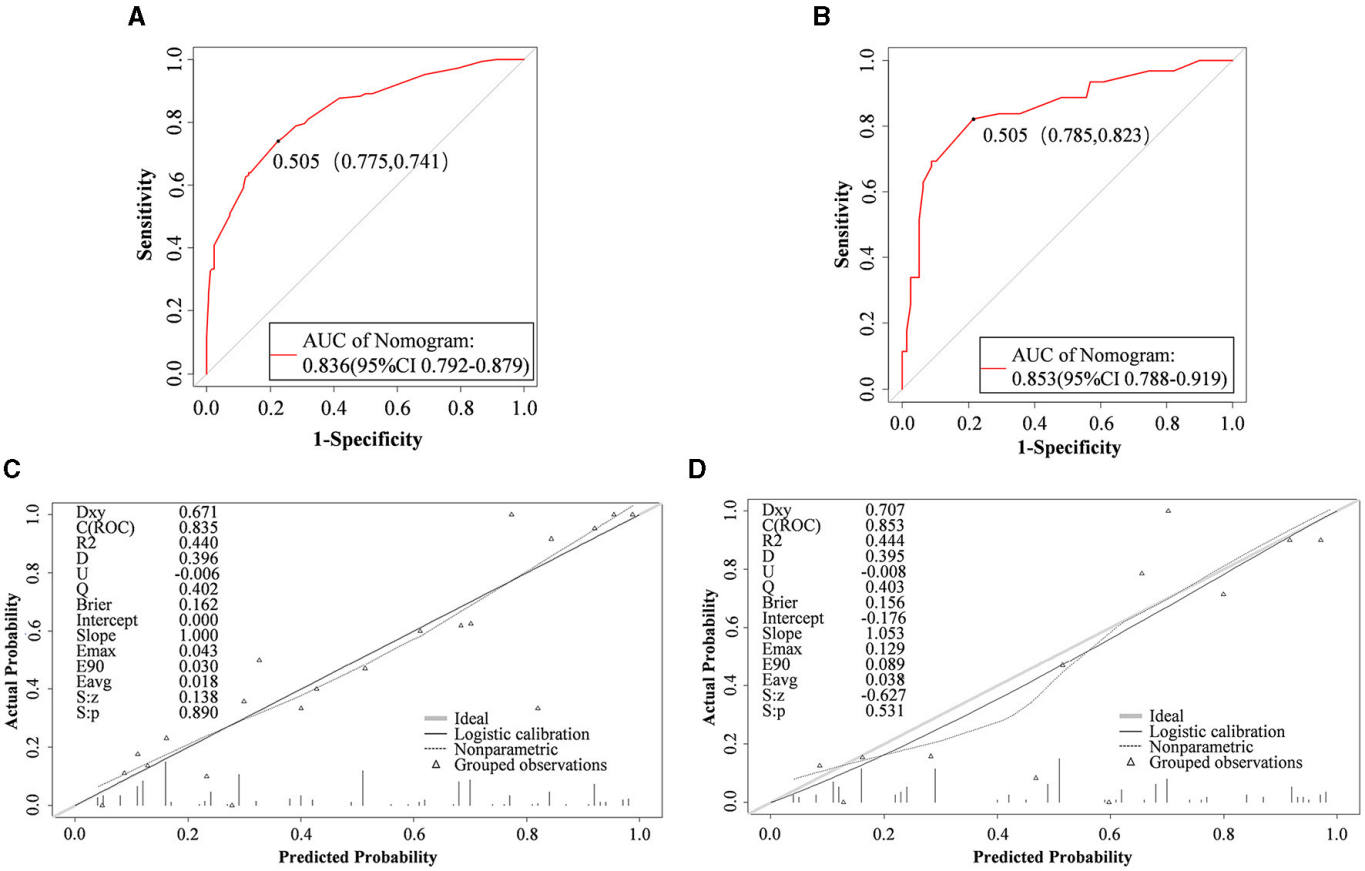
Nomogram performance. ROC curves of the mode for predicting ENFI probabilities in the training cohort **(A)** and validation cohort **(B)**. Calibration plots for predicting ENFI probabilities in the training cohort **(C)** and validation cohort **(D)**. The gray slash represents the ideal reference line, and the black curve represents the predictive performance of the model. The closer the black curve is to the gray slash, the closer the predicted outcome event is to the actual event.

Internal validation of the model was performed on the training set data, and the area under the ROC curve (AUC) was 0.836 (95% CI: 0.792–0.879), as shown in Figure 4A, thus indicating that the model had a good degree of differentiation. The probability of the model's critical value (maximal Jordon's index) was 0.505, a value exceeding it enabling identification of patients at high risk of early ENFI after gastric cancer resection. Correspondingly, the sensitivity of the model is 0.775 and the specificity is 0.741. The calibration curve revealed that the trend of the risk curve of early ENFI after gastric cancer resection predicted by the column-line graph model was essentially the same as that of the actual risk curve (Figure 4C), thus suggesting that the model had a good predictive efficacy. The Hosmer-Lemeshow test showed χ ^2^ = 4.368 (*P* = 0.737), thus indicating the model's good calibration.

#### 3.4.2 External validation

External validation of the model was performed on the validation set data, and indicating the AUC was 0.853 (95% CI: 0.788–0.919) (Figure 4B). The calibration curves showed that the trend of the risk curve of early ENFI occurrence after gastric cancer resection predicted by the column-line graph model simulation was essentially the same as that of the risk curve of actual occurrence (Figure 4D). The Hosmer-Lemeshow test indicated χ^2^ = 13.740 (*P* = 0.089).

## 4 Discussion

### 4.1 The incidence of early ENFI in patients undergoing gastric cancer resection is higher than that in other surgical diseases and has specific predictors

The burden of gastric cancer in China is severe, and clinical data in recent years have shown that the incidence and death rates for gastric cancer are the third highest among all tumors in China (16). Relevant studies (1) have reported that patients with gastric cancer are often malnourished after surgical treatment, thus further increasing the incidence of postoperative complications and mortality in patients. Early initiation of enteral nutrition after gastric cancer resection can improve intestinal function, protect the intestinal mucosal barrier, and decrease the incidence of malnutrition (1), and these effects are clinically important in improving patient prognosis. No gold standard exists for the definition of early nutritional support, but Enhanced Recovery After Surgery (ERAS) (17) suggests that enteral nutritional support should be administered to patients within 24 h after surgery. This study defined ENFI occurring within 1–3 d after gastric cancer surgery as early postoperative enteral nutrition intolerance. ENFI is a group of clinical syndromes including diarrhea, abdominal distension, constipation, vomiting, high gastric residual volume, interruption of enteral nutrition, and low feeding volume during the process of enteral nutrition (18). The main causes of ENFI differ among diseases. For example, patients with hemorrhagic stroke (19) usually show intestinal dyskinesia; patients with severe acute pancreatic cancer (20) typically have impaired intestinal function due to extravasation of pancreatic fluid; and patients with severe burns (21) tend to have ischemia, edema, and erosion of the gastrointestinal mucosa. Therefore, variability exists among the core predictors associated with different diseases. The core predictors of early ENFI after gastric cancer resection are closely related to early postoperative gastrointestinal function impairment, but no uniform standard for gastrointestinal function impairment exists, and this impairment is difficult to quantitatively assess. Therefore, the predictors closely associated with gastrointestinal dysfunction are of great significance in the prediction of early ENFI after gastric cancer resection. These predictors include intra-abdominal pressure, surgical procedure, and the type of enteral nutritional solution. A total of 470 patients with gastric cancer were included in this study, and the incidence rate of postoperative early ENFI was 44.47%, a value higher than other surgical patients, in agreement with the results of a previous study (10). Our findings further indicated that the incidence rate of postoperative early ENFI in gastric cancer was high. The results of this study demonstrated that early ENFI in patients after gastric cancer resection occurred on average 2.27 days after the implementation of enteral nutrition therapy. These findings occurred primarily because patients were given 5% GNS/0.9% NaCl through tube feeding on the first day after gastric cancer surgery in our study center to pre-adapt the intestines to digestion of nutrient solution, and intolerance symptoms often began to appear on the 2^nd^ day after administration of enteral nutrient solution. Abdominal distension, the main symptom, accounted for 72% of all symptoms. Therefore, focus should be placed on monitoring and preventing abdominal bloating symptoms in days 1–3 after implementation of enteral nutrition, to ensure that enteral nutrition can be provided safely and effectively.

### 4.2 Analysis of factors influencing early ENFI in postoperative patients with gastric cancer

Herein, the independent factors influencing early ENFI after gastric cancer resection were found to be the intra-abdominal pressure before enteral feeding, surgical approach, POMS-SF score, Pre-adaptive techniques for preoperative enteral nutrition, and type of enteral nutrition solution.

Intra-abdominal pressure is the earliest and most sensitive reaction to the gastrointestinal tract. Moreover, intra-abdominal pressure dramatically reflects intestinal function (22). A normal intra-abdominal pressure is 5–7 mmHg. The intra-abdominal pressure continually exceeding 12 mmHg is called intra-abdominal hypertension (16). Our findings indicated an incidence of intra-abdominal hypertension after gastric cancer resection of 10.21%, whereas the incidence of early ENFI in patients with intra-abdominal pressure ≥12 mmHg after gastric cancer resection was 6.79 times higher than that in patients with intra-abdominal pressure < 12 mmHg (*P* = 0.003). This finding suggested that intra-abdominal hypertension is a risk factor for early ENFI in patients after gastric cancer resection. A prior study (12) have pointed out that patients with abdominal surgery are at high risk of intra-abdominal hypertension, and gastric cancer resection is a common abdominal surgery. The main cause for the early occurrence of ENFI after gastric cancer surgery is due to anastomotic effusion/bleeding/infection, decreased abdominal wall compliance caused by abdominal wall incision, etc., resulting in low perfusion of tissues and organs. In this study, the incidence of early ENFI in patients who underwent laparoscopic surgery was 2.21 times higher than that in patients who underwent open surgery (*P* = 0.006). This finding was associated with increased abdominal pressure due to the accumulation of air in the abdominal cavity after laparoscopic surgery. Our results suggest that clinical implementation of EN is beneficial in decreasing the ENFI by balancing the relationships among the enteral nutrition infusion rate, intra-abdominal pressure, and intestinal function according to the intra-abdominal pressure value. Previously study (12) supports that intra-abdominal pressure should be measured at least every 4 h after initiation of enteral feeding. When intra-abdominal pressure (IAP) is ≥12 mmHg, intra-abdominal pressure should be measured at least every 4 h after initiating enteral feeding. The infusion rate of enteral nutrition directly affects the patient's abdominal pressure. The recommended starting rate for early enteral nutrition (EEN) is 10–20 ml/h, using an enteral nutrition infusion pump to gradually increase the rate uniformly and incrementally to the target rate, with interruptions minimized as much as possible during this period (23–26). Therefore, constructing an enteral nutrition program guided by intra-abdominal pressure monitoring for patients after gastric cancer resection aids in early achievement of target feeding volume and promotion of patient recovery.

Most patients with tumors experience various types of adverse emotions, such as anxiety, depression, and fear. Changes in emotions can influence the intestines through the brain-gut axis, thereby affecting intestinal function and flora. Therefore, emotional state is closely associated with intestinal function. The POMS-SF can be used for self-assessment of patients' psychological state in the prior week. Our findings indicated that the postoperative ENFI rates in patients with gastric cancer with preoperative POMS-SF ≥27.5 were 5.07 times higher than those in patients with POMS-SF < 27.5 (*P* < 0.001). The cause for this finding may be that the central nervous system work with the gut by neurological and endocrine pathways, and that the sympathetic portion of the autonomic nervous system and the hypothalamo-pituitary-adrenal axis cooperatively regulate secretion, intestinal motility, and blood flow, thereby affecting intestinal permeability and ultimately postoperative enteral nutritional tolerance (27). Similarly, a retrospective study (28) has shown that patients attending psychiatric clinics frequently complain of gastrointestinal symptoms such as constipation, bloating, and abdominal pain, and that 36.5% of patients with gastrointestinal dysfunction also present with psychiatric symptoms. Several studies (29, 30) have examined preoperative psychological pre-habilitation of patients with gastric cancer. The main intervention strategies include cognitive psychological support, attention transfer, muscle relaxation, and training in positive thinking. All the findings have indicated that preoperative psychological adjusting positively influences the prognosis of patients with gastric cancer.

The results of preoperative enteral nutrition pre-adaptive technology for patients with gastric cancer at our research center (31) have indicated lower incidence of abdominal distension, diarrhea, and abdominal pain in the observation group than the control group (*P* < 0.05). Thus, the preoperative implementation of enteral nutrition pre-adaptive technology can decrease the incidence of enteral nutrition-related adverse symptoms in patients who have undergone gastric cancer resection. In the present study, the incidence of ENFI in patients with preoperative implementation of the enteral nutrition pre-adaptation technique was 0.29 (*P* = 0.003) times lower than that of patients who did not receive the technique, which was a protective factor. The role of intestinal pre-adaptation is mainly entry of nutrient solution into the intestinal lumen, through intestinal digestion and absorption of nutrients, and stimulation of the intestinal tract to produce an important growth factor, which in turn stimulates the digestion and absorption of enteral nutrient solution in the postoperative period. Therefore, preoperative enteral nutrition pre-adaptation techniques have positive effects on early postoperative ENFI.

Several studies have demonstrated that short peptide enteral nutrition (SP) can significantly enhance the recovery of intestinal mucosal epithelial function through the following mechanisms. It enhances barrier integrity by upregulating tight junction proteins (32), thereby reducing permeability and bacterial translocation. Additionally, it stimulates endothelial nitric oxide synthase (eNOS) activity, improving microcirculation (33) and facilitating the delivery of essential nutrients required for tissue repair. Immunomodulatory effects include lowering pro-inflammatory cytokine levels (e.g., IL-6, TNF-α) while increasing protective cytokines (e.g., IL-10), creating an anti-inflammatory environment favorable for healing (34). Furthermore, SP composed of smaller peptide chains, it does not require complex digestive processes (35), thus decrease the digestive workload, contributing to overall mucosal recovery. This nutritional solution is suitable for patients with gastrointestinal dysfunction, such as slow gastric emptying and dyspepsia (36). However, because of its absorption without a need for digestive enzymes to break down and prolonged use diminishes digestive function. Therefore, for postoperative patients with gastric cancer with good gastrointestinal function, use of total protein enteral nutrition (TP) is recommended. Herein, the difference between the incidence of ENFI with TP and SP was not statistically significant (*P* > 0.05), similarly to findings reported by Yuhua et al. (37). SP is a pre-digested nutritional solution, which is absorbed faster than total protein with fiber enteral nutrition (TFP). TFP tends to induce intestinal flatulence because of the presence of dietary fiber. The results of this study indicated that the incidence of early postoperative ENFI in patients fed TFP after surgery was 7.23 times higher than in those fed SP (*P* < 0.001). In conclusion, for postoperative gastric cancer patients with good gastrointestinal function, TP should be the first choice for enteral nutrition, followed by SP, while choosing TFP caution the risk of ENFI. For patients with impaired gastrointestinal function, SP formulas should be prioritized.

### 4.3 Early ENFI prediction model with good predictive efficacy in postoperative patients with gastric cancer

In this study, the pre-conducted enteral nutrition pre-adaptation technique was as a predictor, and a risk prediction model of early ENFI after gastric cancer resection was constructed by combining the preoperative emotional state and the level of postoperative intra-abdominal pressure and other statistically significant influencing factors. Some researchers (11) have constructed a prediction model for postoperative ENFI in gastric cancer through logistic regression as follows: ENFI = history of constipation × 3.67 + 3.548 × preoperative ASA score grade III + 3.324 × postoperative pain score ≥4 points at 6 h postoperatively + 1.104 × postoperative WBC on the first day of operation. The model does not account for the influence of intra-abdominal pressure and emotional state on ENFI in postoperative patients with gastric cancer. At present, domestic postoperative pain management adopts multimodal combined analgesia, with a goal of maintaining patients' pain NRS scores at 2–3 points in resting state through drug intervention, and essentially no difference in the pain score at 6 h postoperative among patients. Therefore, the predictive factors of the model must be further optimized. In this study, Lasso regression was used to screen the predictors. This method has a strong ability to automatically select important features and to address problems of multicollinearity, thus improving the generalization ability of the model and avoid overfitting. The AUCs in internal and external validation of the model of 0.836 and 0.853, respectively, indicated the model's good predictive ability. The calibration curves were consistent with the trend of the ideal curve, thus demonstrating the model's good differentiation and calibration. The ENFI prediction model for patients undergoing gastric cancer resection, developed from a training set of 329 cases, yielded a Youden index of 0.505. This finding suggests that in clinical practice, a predicted probability exceeding 0.505 can classify a patient as high-risk for ENFI, thereby providing a basis for guiding subsequent nutritional intervention strategies.

## 5 Conclusion

In summary, ENFI is a common complication during early enteral nutrition in patients after gastric cancer resection, and effective prediction of patients' tolerance or not is key to the smooth implementation of early enteral nutrition. The ENFI risk prediction model constructed in this study is innovative in that it fully considers the effects of intra-abdominal pressure and preoperative mental state. The pre-adaptive technology of enteral nutrition at our center in early stages after gastric cancer resection was used as a predictor, which showed good predictive efficacy. Our model may provide guidance for screening of high-risk groups for ENFI and formulation of intra-abdominal pressure-based enteral nutrition infusion proposal after gastric cancer surgery. However, the present study has several limitations, in that it was a single-center study and could not be validated in an external gastric cancer cohort. In the future, results from multi-center studies will be necessary to further optimize the model and improve its generalization ability.

## Data Availability

The original contributions presented in the study are included in the article/supplementary material, further inquiries can be directed to the corresponding authors.
